# One-year outcomes following surgery for necrotising enterocolitis: a UK-wide cohort study

**DOI:** 10.1136/archdischild-2017-313113

**Published:** 2017-11-01

**Authors:** Benjamin Saul Raywood Allin, Anna-May Long, Amit Gupta, Kokila Lakhoo, Marian Knight, The British Association of Paediatric Surgeons Congenital Anomalies Surveillance System Necrotising Enterocolitis Collaboration

**Affiliations:** 1 National Perinatal Epidemiology Unit, University of Oxford, Oxford, UK; 2 Department of Paediatric Surgery, Oxford Children’s Hospital, Oxford, UK; 3 Neonatal Intensive Care Unit, Oxford Children’s Hospital, Oxford, UK

**Keywords:** necrotising enterocolitis, paediatric surgery, gastroenterology, neonatology

## Abstract

**Objective:**

The objective was to describe outcomes and investigate factors affecting prognosis at 1 year post intervention for infants with surgical necrotising enterocolitis (NEC).

**Design:**

Using the British Association of Paediatric Surgeons Congenital Anomalies Surveillance System, we conducted a prospective, multicentre cohort study of every infant reported to require surgical intervention for NEC in the UK and Ireland between 1 March 2013 and 28 February 2014. Association of independent variables with 1-year mortality was investigated using multivariable logistic regression analysis.

**Setting:**

All 28 paediatric surgical centres in the UK and Ireland.

**Patients:**

Infants were eligible for inclusion if they were diagnosed with NEC and deemed to require surgical intervention, regardless of whether that intervention was delivered.

**Outcomes:**

Primary outcome was mortality within 1 year of the decision to intervene surgically.

**Results:**

236 infants were included in the study. 208 (88%) infants had 1-year follow-up. 59 of the 203 infants with known survival status (29%, 95% CI 23% to 36%) died within 1 year of the decision to intervene surgically. Following adjustment, key factors associated with reduced 1-year mortality included older gestational age at birth (adjusted OR (aOR) 0.87, 95% CI 0.78 to 0.96). Being small for gestational age (SGA) (aOR 3.6, 95% CI 1.4 to 9.5) and requiring parenteral nutrition at 28 days post-decision to intervene surgically (aOR 3.5, 95% CI 1.1 to 11.03) were associated with increased 1-year mortality.

**Conclusions:**

Parents of infants undergoing surgery for NEC should be counselled that there is approximately a 1:3 risk of death in the first post-operative year but that the risk is lower for infants who are of greater gestational age at birth, who are not SGA and who do not require parenteral nutrition at 28 days post-intervention.

**What is already known on this topic?**

- Five per 1000 infants admitted to neonatal units are diagnosed with necrotising enterocolitis (NEC), and one in four of those infants require surgical intervention.
- Surgical NEC is a major cause of early morbidity and mortality.
- More nuanced data are required from high-quality studies to allow accurate counselling of parents of infants with NEC.

**What this study adds?**

- Approximately one in three infants with NEC die within the first postoperative year, and 6 in 10 require at least one additional operation.
- Older gestational age at birth, older age at presentation with symptoms of NEC and more limited disease are associated with lower odds of 1-year mortality.
- Being small for gestational age, requiring inotropes at time of surgical intervention and requiring parenteral nutrition at 28 days post-decision to intervene surgically are associated with a raised odds of 1-year mortality.

## Background

Necrotising enterocolitis (NEC) affects approximately 1 per 1000 live-born infants,^1^ with approximately one in four infants with NEC undergoing surgical intervention.^2^ NEC thus represents one of the major neonatal gastrointestinal emergencies and is a significant contributor to rates of neonatal mortality.^3 4^ A constant message from the parent and patient group advising our research programme is that the advice and counselling they receive varies significantly dependent on the clinician they are talking to or the hospital they visit. Such variation in advice is a cause of great distress, and it is likely that this variation occurs due to a lack of relevant, nuanced, large-scale data on which to base parental counselling.^5^

The aim of this prospective nationwide cohort study was therefore to provide detailed information relating to outcomes in the first postoperative year and factors that affect prognosis that could subsequently form a resource from which to counsel parents of children undergoing surgical intervention for NEC. Outcomes within the first 28 postoperative days have been previously published elsewhere.^2^

## Methods

### Study design

Using the British Association of Paediatric Surgeons Congenital Anomalies Surveillance System, we conducted a prospective, multicentre cohort study of every infant reported to require surgical intervention for NEC in the UK and Ireland between 1 March 2013 and 28 February 2014.

Cases were identified via monthly reporting cards sent to lead clinicians at all 28 paediatric surgical centres in the UK and Ireland, with detailed data collection forms completed in response to notification of a case and again 1 year later. Data handling was as described in previous publications.^6^

### Case definition and subgroup analysis

Infants were eligible for inclusion in the study if they were deemed to require surgical intervention for NEC, regardless of whether that intervention was actually delivered. NEC could be diagnosed in three different ways dependent on the pathway an infant followed:

1. Clinically using the Vermont Oxford Network Criteria^7^ for infants where necessity for surgical intervention was confirmed but no laparotomy or postmortem undertaken to allow confirmation of NEC on visual inspection of the bowel.
2. At the time of postmortem (when undertaken), for infants where surgical intervention was deemed necessary for a presumptive diagnosis of NEC but no laparotomy was performed to allow confirmation of NEC on visual inspection of the bowel.
3. On visual inspection of the bowel at time of laparotomy.

Surgical intervention refers to both laparotomy and primary peritoneal drainage and is used in this manner throughout the manuscript.

Infants who at the time of laparotomy were deemed to have a cause for their symptoms other than NEC were excluded from the analysis. However, because there is evidence to suggest that the differences in presentation between SIP and NEC are too small to allow reliable differentiation prelaparotomy^8^ and there exists significant debate as to whether SIP and NEC represent two separate conditions or different end-points of the same underlying pathway, the case definition has deliberately been designed to encompass both entities. Previous work from the Vermont Oxford Network has however suggested that mortality is different for infants with laparotomy-confirmed SIP than it is for infants with laparotomy-confirmed NEC.^8^ In order to present data in the most clinically appropriate manner, we have therefore elected to combine the two entities for the purposes of preoperative prognostication, but following operative confirmation of diagnosis, to examine our primary outcome, mortality, separately for those infants with confirmed NEC and those with confirmed SIP.

### Outcomes

Our primary outcome of interest was mortality within 1 year of the decision to intervene surgically. Secondary outcomes included further surgical intervention and discharge home in the first year after the decision to intervene surgically and parenteral nutrition use, intestinal failure associated liver disease (IFALD) and liver transplant at 1 year post-decision to intervene surgically. IFALD is defined as per the 2009 review of current management practices in IFALD conducted by the British Society of Paediatric Gastroenterology, Hepatology and Nutrition and previously used by Bishay *et al* 9:

- Type 1 (early) IFALD—persistent elevation of alkaline phosphatase for 6 weeks or longer.
- Type 2 (established)—additional elevated total bilirubin (>50 μmol/L).
- Type 3 (late)—additional clinical signs of end-stage liver disease.

### Mortality estimates

Actual mortality rates were calculated based on the returned data. Minimum/maximum estimates of mortality were calculated on the assumption that all infants who were lost to follow-up had survived/died, respectively.

In order to allow a more direct comparison with the Vermont Oxford Network studies, which only include infants with a birth weight of less than 1500 g, a subgroup analysis of this cohort was undertaken.

### Statistical analysis

Rates for each of the primary and secondary outcome measures were calculated based on the number of infants with information returned for that variable.

ORs and 95% CIs were calculated for independent variables considered likely to have an impact on mortality at 1 year post-decision to intervene surgically (online supplementary information 1). Two multivariable logistic regression analysis models were then developed. The first was designed to investigate the association of key preoperative factors with 1-year mortality, while the second was designed to assess the association of operative findings and early markers of morbidity with 1-year mortality.

10.1136/archdischild-2017-313113.supp1 Supplementary file 1

There is existing evidence of an association between mortality and both gestational age and being small for gestational age. An a priori decision was therefore made to include both of these variables in the model, regardless of the statistical significance of their association with mortality. Small for gestational age was defined as a birth weight of <10th centile for gestational age, with centiles calculated from the infant’s birth weight z-score. Other variables were assessed for inclusion in the model in a forward stepwise manner in order of statistical significance if they were shown on univariable analysis to be associated with 1-year mortality, as defined by a p value of <0.1. Independent variables were dropped from the model if they did not statistically significantly affect the fit of the model, as defined by a p value of >0.05 on likelihood ratio testing.

The second multivariable model was developed using the first as a base, with operative findings and markers of early postoperative morbidity assessed for inclusion in the model in addition to those retained in the preoperative model. Because of existing evidence that infants diagnosed with SIP at the time of laparotomy may have different outcomes to those with NEC,^8^ an a priori decision was made to include definitive diagnosis of SIP in the model regardless of its association with 1-year mortality on univariable analysis. Other operative findings and markers of early postoperative morbidity were assessed for inclusion in the model in the same manner as independent variables in the preoperative model. Complete case analysis was used in both models.

## Results

### Follow-up and demographics

Two hundred and thirty-six infants were included in the study, with 1-year follow-up information obtained for 208 (88%). Thirty-two infants with complete follow-up (16%) and none who were lost to follow-up had a definitive diagnosis of SIP (p=0.02). There were no other statistically significant differences between infants with and without follow-up (table 1).

**Table 1:** Baseline demographics and predictors of 1-year mortality in infants with complete follow-up and infants lost to follow-up

Baseline characteristic*		Infants lost to follow-up n (%) n=28	Infants with 1-year follow-up n (%) n=208	p Value
Ethnicity	White British	11 (42%)	114 (59%)	0.10
	Other	15 (58%)	78 (41%)	
Sex	Male	16 (57%)	129 (62%)	0.62
	Female	12 (43%)	79 (38%)	
Median (IQR) gestational age at birth (completed weeks)		27 (26–33)	26 (25–30)	0.12
Median (IQR) birth weight (grams)		1013 (657–1165)	907 (720–1310)	0.15
Small for gestational age (<10th centile)	Yes	6 (22%)	30 (15%)	0.30
	No	21 (78%)	176 (85%)	
Ligation of PDA	Yes	3 (11%)	15 (7%)	0.48
	No	24 (89%)	192 (93%)	
Non-cardiac congenital anomaly	Yes	3 (11%)	23 (11%)	1.00
	No	24 (89%)	184 (89%)	
Abdominal wall erythema or discolouration at presentation	Yes	7 (25%)	65 (31%)	0.5
	No	21 (75%)	143 (69%)	
Operative diagnosis of SIP†	Yes	0 (0%)	32 (16%)	0.02
	No	27 (100%)	162 (84%)	
Surgical procedure	Resection and primary anastomosis	5 (19%)	35 (17%)	0.83
	Resection and stoma formation	16 (62%)	105 (51%)	
	Stoma without resection	4 (15%)	34 (16%)	
	Clip and drop with resection	1 (4%)	9 (4%)	
	Open and close laparotomy	0	10 (5%)	
	Negative laparotomy	0	2 (1%)	
	Drain only	0	6 (3%)	
	No surgical intervention	0	6 (3%)	

*Complete data were not obtained for all characteristics for all infants, and therefore numbers and percentages presented, including for surgical procedure, are based on the number of infants with data returned for that characteristic.

†Diagnosis was not possible for 12 infants who did not undergo laparotomy and was unknown in three despite undergoing a laparotomy.

PDA, patent ductus arteriosis, SIP, spontaneous intestinal perforation

### Mortality

Fifty-nine (29%) of the 203 infants with a known 1-year survival status had died within a year of the decision to intervene surgically. Minimum and maximum estimates of mortality are 25% and 39%, respectively (table 2). Of the 59 infants who died, 43 (73%) did so prior to 28 days post-decision to intervene surgically and 16 (27%) between 28 days and 1 year post-decision to intervene surgically. The median time (IQR) from decision to intervene surgically until death was 8 days (1–44 days).

**Table 2:** Mortality rates

	Number of infants	Survival status at 1 year post-decision to intervene surgically			One-year mortality rates	
		Unknown n (%)	Died n (%)	Survived n (%)	n (%, 95% CI)	Estimate of min to max
Entire cohort	236	33 (14%)	59 (25%)	144 (61%)	59/203 (29%, 23% to 36%)	25%–39%
Confirmed NEC*	189	30 (16%)	41 (22%)	118 (62%)	41/169 (24%, 18% to 31%)	22%–38%
Confirmed SIP*	32	0 (0%)	11 (34%)	21 (66%)	11/32 (34%, 19% to 53%)	34%–34%
Laparotomy performed but unknown final diagnosis*	3	2 (66%)	0	1 (33%)	N/A	
No laparotomy performed	12	1 (8%)	7 (58%)	4 (33%)	7/11 (64%, 31% to 90%)	58%–67%
Infants ≤1500 g	189	26 (14%)	50 (26%)	113 (60%)	50/163 (31%, 24% to 38%)	26%–40%

*Data only available for the 224 infants who underwent laparotomy.

NEC, necrotising enterocolitis.

Of the 163 infants with known 1-year survival status and a birth weight of <1500 g, 50 (31%) had died prior to 1 year. Minimum estimated mortality was 26% and maximum estimated mortality was 40% (table 2 and table 3).

**Table 3:** One-year mortality rates by gestational age

Gestational age at birth (completed weeks)	Number of infants	Number of infants missing 1-year mortality data n (%)*	Infants died n (%), 95% CI
<26	84	6 (7%)	26 (33%), 23 to 45
26–27	54	11 (20%)	12 (28%), 15 to 44
28–31	54	8 (15%)	13 (28%), 16 to 43
32–36	26	4 (15%)	7 (32%), 14 to 55
≥37	17	3 (18%)	1 (7%), 0.2 to 34

*One infant is missing gestational age at birth and 1-year mortality data.

### Cause of death

The causes of death of infants prior to 28 days post-decision to intervene surgically have been previously reported.^2^ Of the 16 infants who died between 28 days and 1 year post-decision to intervene surgically, seven (43%) died as a direct result of NEC, three (19%) died as a result of extreme prematurity, one (6%) died from multiorgan failure, one (6%) died from circulatory collapse of unknown cause, one (6%) died as a result of massive intracerebral haemorrhage, one (6%) died from liver failure and two (13%) had unknown causes of death.

### Further surgical intervention

Of the 196 infants with 1-year follow-up and information recorded, there were 68 infants (35%) who had one further procedure, 31 infants (16%) who had two further procedures and 19 infants (10%) who had three or more further procedures. Forty-six infants in the cohort (23%) were confirmed to have undergone at least one further laparotomy (table 4). At the time of laparotomy, 24 infants (13%) had a further resection for active NEC, while 17 infants (9%) underwent formation of a new stoma. The most common additional procedure overall, however, was stoma closure, which was undertaken in 83 infants (42%). Sixty-six stoma closures (80%) were performed electively in order to restore gut continuity, and these were performed at a median of 98 days post initial surgical intervention (IQR 52–148 days). There were a further 12 infants (15%) who had their stoma closed due to high output, and three (4%) in whom stoma closure was deemed more appropriate than stoma revision. The indication for stoma closure in two infants (1%) was unknown. Other common additional procedures included vascular access (21 infants, 10%), line removal (8 infants, 4%) and inguinal herniotomy (11 infants, 5%).

**Table 4:** Other outcomes at 1 year post-decision to intervene surgically

Outcome		Number of infants (%)
Number of additional operations* n=236 Missing data for 40 infants (17%)	0	78 (40%)
	1	68 (35%)
	2	31 (16%)
	3 or more	19 (10%)
IFALD† N=177 Missing data for 34 infants (19%)	None	128 (90%)
	Type 1	5 (3%)
	Type 2	9 (6%)
	Type 3	1 (1%)
Parenteral nutrition dependency† N=177 Missing data for 35 infants **(20%)**	No	130 (92%)
	Yes	12 (8%)
Liver transplant† N=177 Missing data for 35 infants **(20%)**	No	141 (99%)
	Yes	1 (1%)
Discharge home‡ n=177 Missing data for 33 infants **(19%)**	No	3 (2%)
	Yes	126 (88%)
	Unclear	15 (10%)

*Percentage of infants with follow-up data available for outcome.

†Percentage of infants who had not died prior to 1 year post-decision to intervene surgically and with follow-up data available for outcome.

‡Percentage of infants who had not died prior to 1 year post-decision to intervene surgically.

### Intestinal and hepatic function

Of the 143 infants who were alive at 1 year post-decision to intervene surgically with information returned in relation to IFALD, 5 (3.5%) had been diagnosed with type 1 (early) IFALD, 9 (6%) with type 2 (established) and 1 (1%) with type 3 (late). There were also 12 infants (8%) out of the 142 alive with returned information who required ongoing parenteral nutritional support at 1 year post-decision to intervene surgically and one infant (1%) who had undergone a liver transplant within a year of the decision to intervene surgically (table 4).

### Discharge home

Of the 144 infants who were known to be alive at 1 year post-decision to intervene, 126 (88%) were confirmed to have been discharged home, while three (2%) remained as inpatients. Discharge status was unknown in 15 (10%) (table 4).

### Preoperative factors associated with 1-year mortality

The following variables were assessed for inclusion in the multivariable model due to the statistical significance of their association with mortality on univariable analysis (p≤0.1): ethnicity, non-cardiac congenital anomaly, abdominal wall erythema or discolouration at presentation, inotropes at presentation, inotropes required at time of surgical intervention, ventilated at presentation, ventilated at time of surgical intervention, age at presentation to the treating hospital with first symptoms of NEC, at least one region of colon unaffected by NEC and need for parenteral nutrition at 28 days post-decision to intervene surgically. Results of the univariable analysis can be found in online supplementary table S1.

Following adjustment for factors retained in the preoperative multivariable model (table 5), increasing gestational age at birth (adjusted OR (aOR) 0.87, 95% CI 0.78 to 0.96, p=0.007) and increasing age at presentation to the treating hospital with first symptoms of NEC (aOR 0.97, 95% CI 0.95 to 0.99, p=0.007), were significantly associated with a lower odds of 1-year mortality. Being small for gestational age at birth (aOR 3.6, 95% CI 1.4 to 9.5, p=0.008), requiring inotropes at the time of surgical intervention (aOR 2.7, 95% CI 1.3 to 5.5, p=0.006) and a diagnosis of a non-cardiac congenital anomaly (aOR 6.2, 95% CI 1.8 to 21.4, p=0.004) were all associated with a raised odds of 1-year mortality.

**Table 5:** Preoperative characteristics associated with 1-year mortality following multivariable logistic regression analysis

Independent variables associated with a lower odds of mortality at 1 year post-decision to intervene surgically		
Preoperative characteristic	OR (95% CI)	aOR (95% CI)
Gestational age at birth (per completed week increase)	0.9 (0.88 to 1.02) p=0.15	0.87 (0.78 to 0.96) p=0.007
Age at presentation to the treating hospital with first symptoms of NEC (per 1 day increase)	0.98 (0.96 to 1.0) p=0.06	0.97 (0.95 to 0.99) p=0.007

Independent variables associated with a raised odds of mortality at 1 year post-decision to intervene surgically					
Preoperative characteristic		Died n (%) n=59	Alive n (%) n=149	OR (95% CI)	aOR (95% CI)
Small for gestational age at birth	Yes	14 (25%)	16 (11%)	2.6 (10.7 to 6.2) p=0.02	3.6 (1.4 to 9.5) p=0.008
	No	43 (75%)	128 (89%)		
Inotropes required at time of decision for surgical intervention*	Yes	32 (54%)	43 (30%)	2.8 (1.4 to 5.4) p=0.001	2.7 (1.3 to 5.5) p=0.006
	No	27 (466%)	100 (70%)		
Non-cardiac congenital anomaly*	Yes	10 (17%)	13 (9%)	2.0 (0.8 to 5.6) p=0.1	6.2 (1.8 to 21.4) p=0.004
	No	48 (83%)	131 (91%)		

Additionally adjusted for the presence of abdominal wall erythema or discolouration at the time of presentation, which was retained in the model but was not statistically significantly associated with 1-year mortality.

*Based on number of infants with complete information for variable.

NEC, necrotising enterocolitis.

### Association of operative findings and early morbidity with 1-year mortality

Results of the univariable analysis can be found in online supplementary table S1. Following adjustment for those variables retained in the preoperative model and additional operative/early postoperative findings, two factors were identified as having an association with 1-year mortality. Identification of at least one region of colon that was unaffected by NEC at the time of operation was associated with a significantly lower odds of 1-year mortality (aOR 0.01, 95% CI 0.0007 to 0.19, p=0.002), while the need for parenteral nutritional support at 28 days post-decision to intervene surgically was associated with a significantly raised odds of mortality in the first year after the decision to intervene surgically (aOR 3.5, 95% CI 1.1 to 11.0, p=0.03) (table 6).

**Table 6:** Association of operative findings and early postoperative outcomes with 1-year mortality

Operative findings and early morbidity		Died n (%)	Alive n (%)	OR (95% CI)	aOR (95% CI)*
Definitive diagnosis of SIP at laparotomy	Yes	11 (21%)	21 (15%)	1.5 (0.6 to 3.6) p=0.3	1.1 (0.4 to 3.4) p=0.8
	No	41 (79%)	118 (85%)		
At least one region of colon unaffected by NEC	Yes	43 (86%)	137 (99%)	0.04 (0.001 to 0.4) p=0.0001	0.01 (0.0007 to 0.19) p=0.002
	No	7 (14%)	1 (1%)		
Need for parenteral nutritional support at 28 days post-decision to intervene	Yes	52 (88%)	90 (62%)	4.5 (1.8 to 12.4) p=0.0003	3.5 (1.1 to 11.03) p=0.034
	No	7 (12%)	54 (38%)		

*Adjusted for factors retained in preoperative model in addition to factors included in operative findings and early morbidity model.

NEC, necrotising enterocolitis.

## Discussion

This nationwide cohort study of infants with surgical NEC has estimated 1-year mortality at 29%, 1 year parenteral nutrition dependence at 9% and that 61% of infants had additional surgical intervention in the first postoperative year. Forty-three per cent of infants who died between 28 days and 1 year post-decision to intervene surgically died as a direct result of NEC. These outcomes highlight the ongoing burden of disease associated with surgical NEC, even outside of the initial postoperative period.

The nuanced data collected in this study and the low loss-to-follow-up rate have allowed an accurate description of the burden of disease associated with surgical NEC on a national basis. By capturing robust information relating to risk factors, operative technique and both early and late outcomes from a national cohort of infants, the results of this study can be used for accurate benchmarking of outcomes and counselling of parents. This study is, however, open to the limitations of all observational studies, including confounding from unmeasured variables. More specific limitations include the fact that the threshold for surgical intervention will vary in different centres and that different clinicians will have different thresholds for starting and stopping parenteral nutrition, enteral feeds and inotropes. Such variation in practice combined with the relatively limited numbers in the study, despite its nationwide design and year-long data collection, mean that we may have failed to identify associations between important independent variables and 1-year mortality.

The largest existing studies of infants with NEC are those from the Vermont Oxford Network based in the USA. In these studies, a mortality rate of 35% has been reported for infants with surgically managed NEC with a birth weight of less than 1500 g.^10^ When our study is limited to infants with a birth weight of less than 1500 g, a mortality rate of 31% is seen, which is not statistically significantly different from the 35% reported by the Vermont Oxford Network. Battersby *et al* 11 have recently reported results from an English cohort study investigating incidence and feeding antecedents of severe NEC. In this, they report 139 deaths (33%) among 423 infants with laparotomy-confirmed NEC. There is some overlap in the time period and geographical region of data collection between their study and ours, and the similar incidences of laparotomy confirmed NEC, and lack of a statistically significant difference in mortality rates between the two studies (p=0.1), despite the different study designs, adds reassurance to the robustness of the data presented.

This study provides information to improve the counselling of parents of infants who undergo surgical intervention for NEC. It is important to convey to parents that despite there being approximately a one in three chances of death in the first postoperative year for infants requiring surgical intervention for NEC in the UK, the risk is reduced in infants with a greater gestational age at birth, those whose birth weight is above the 10th centile for their gestational age and those who are older at presentation with symptoms of NEC. Data from the study can also be used to counsel parents that 50% of deaths occur in the first 8 days postoperatively and that if their child survives to 28 days post-decision to intervene surgically and is no longer requiring parenteral nutrition that they have a very good chance of survival to 1 year post-decision to intervene surgically. As well as conveying information related to mortality, parents can be told that nearly two-thirds of infants will require at least one further operation in the first year and that IFALD and parenteral nutrition dependence occur in roughly 1 in 10 infants who are alive at 1 year post-decision to intervene surgically.

We believe that the identified association of 28-day parenteral nutrition requirement and mortality may be due to a combination of three factors. First, that infants who have more severe NEC will be more likely to require parenteral nutrition at 28 days post-decision to intervene surgically than those who are more stable and that the increased odds of mortality is due to the underlying disease severity, not the parenteral nutrition requirement. Second, that parenteral nutrition requirement at 28 days post-decision to intervene surgically is a surrogate marker for intestinal failure and that complications resulting from the intestinal failure are responsible for the increased 1-year mortality, and third, that the increased mortality is occurring as a result of complications secondary to long-term parenteral nutrition use. Regardless of the reason for the association, we believe that it is useful in the counselling of parents to have a reliably defined, easily identifiable marker of morbidity that can be used as a framework around which to discuss potential longer term outcomes.

While this work has provided further information that can be used for counselling of parents, it has also highlighted many further questions. Potentially the most important is how to determine the appropriate point to intervene surgically in infants with NEC. This study has provided evidence that those infants who have more extensive disease and are less clinically stable at the time of laparotomy are less likely to survive to 1 year post-decision to intervene surgically. Investigating methods of robustly diagnosing NEC and identifying the need for surgical intervention prior to infants reaching this point of clinical instability is therefore essential in order to improve outcomes. Conducting a large-scale, international, multicentre cohort study would help to build on work by Battersby *et al* 12 to refine a definition for NEC and identify prognostic factors. Population definition and risk stratification through such a study would lay the groundwork for randomised controlled trials to later compare different methods and timings of surgical intervention. Through work such as this, it would be possible to address the concerns of the National Confidential Enquiry into Patient Outcomes and Death^13^ that ‘difficulty in decision making during both medical management and the decision to operate in babies with NEC’ is a key factor in limiting outcomes.

## References

[1] Holman RC, Stoll BJ, Curns AT, et al Necrotising enterocolitis hospitalisations among neonates in the United States. Paediatr Perinat Epidemiol 2006;20:498–506. 10.1111/j.1365-3016.2006.00756.x 17052286

[2] Allin B, Long AM, Gupta A, et al A UK wide cohort study describing management and outcomes for infants with surgical Necrotising Enterocolitis. Sci Rep 2017;7:41149 http://www.nature.com/articles/srep41149#supplementary-information 10.1038/srep41149 28128283 PMC5269581

[3] Fitzgibbons SC, Ching Y, Yu D, et al Mortality of necrotizing enterocolitis expressed by birth weight categories. J Pediatr Surg 2009;44:1072–6. 10.1016/j.jpedsurg.2009.02.013 19524719

[4] Neu J, Walker WA Necrotizing enterocolitis. N Engl J Med 2011;364:255–64. 10.1056/NEJMra1005408 21247316 PMC3628622

[5] Allin B, Aveyard N, Campion-Smith T, et al What evidence underlies clinical practice in paediatric surgery? a systematic review assessing choice of study design. PLoS One 2016;11:e0150864 10.1371/journal.pone.0150864 26959824 PMC4784961

[6] Owen A, Marven S, Johnson P, et al Gastroschisis: a national cohort study to describe contemporary surgical strategies and outcomes. J Pediatr Surg 2010;45:1808–16. 10.1016/j.jpedsurg.2010.01.036 20850625

[7] Fitzgibbons SC, Ching Y, Yu D, et al Mortality of necrotizing enterocolitis expressed by birth weight categories. J Pediatr Surg 2009;44:1072–6. 10.1016/j.jpedsurg.2009.02.013 19524719

[8] Fisher JG, Jones BA, Gutierrez IM, et al Mortality associated with laparotomy-confirmed neonatal spontaneous intestinal perforation: a prospective 5-year multicenter analysis. J Pediatr Surg 2014;49:1215–9. 10.1016/j.jpedsurg.2013.11.051 25092079

[9] Bishay M, Pichler J, Horn V, et al Intestinal failure-associated liver disease in surgical infants requiring long-term parenteral nutrition. J Pediatr Surg 2012;47:359–62. 10.1016/j.jpedsurg.2011.11.032 22325390

[10] Hull MA, Fisher JG, Gutierrez IM, et al Mortality and management of surgical necrotizing enterocolitis in very low birth weight neonates: a prospective cohort study. J Am Coll Surg 2014;218:1148–55. 10.1016/j.jamcollsurg.2013.11.015 24468227

[11] Battersby C, Longford N, Mandalia S, et al Incidence and enteral feed antecedents of severe neonatal necrotising enterocolitis across neonatal networks in England, 2012-13: a whole-population surveillance study. Lancet Gastroenterol Hepatol 2017;2:43–51. 10.1016/S2468-1253(16)30117-0 28404014

[12] Battersby C, Longford N, Costeloe K, et al Development of a gestational age-specific case definition for neonatal necrotizing enterocolitis. JAMA Pediatr 2017;171:256–63. 10.1001/jamapediatrics.2016.3633 28046187

[13] Mason DW, Gough K, Lucas MJ, et al National Confidential Enquiry into Patient Outcomes and Death (NCEPOD); Are we there yet- a review of organisational and clinical aspects of children’s surgery. London 2011.

