# Supplementary material for: One-year outcomes following surgery for necrotising enterocolitis: a UK-wide cohort study
Source: Arch Dis Child Fetal Neonatal Ed. 2017 Nov 1;103(5):F461–6. doi: 10.1136/archdischild-2017-313113 (PMC6109245; doi:10.1136/archdischild-2017-313113)
Supplement: Supplementary file 1 [file fetalneonatal-2017-313113supp001.docx]

**Supplementary information 1 – Independent risk factors assessed in univariable analysis**

Antenatal/birth factors considered were; ethnicity, gender, gestational age at birth, small for gestational age (as defined by a birth weight of less than the 10^th^ centile for gestational age), plurality, mode of delivery, antenatal corticosteroid administration, and maternal use of indomethacin. Neonatal risk factors considered were; APGAR score less than 5 at ten minutes, umbilical catheter ever used, presence of a patent ductus arteriosus (PDA), indomethacin for PDA closure, PDA ligation, other cardiac surgery, non-cardiac congenital anomaly, antacid use at presentation, whether the infant had ever been enterally fed, if the infant was formula fed at diagnosis, and blood transfusion within two weeks of diagnosis of NEC. Presentation factors considered were; abdominal wall erythema/discolouration at presentation, inotropes at presentation, inotropes at the time of surgical intervention, ventilation at presentation, ventilation at the time of surgical intervention, and radiological evidence of perforation prior to laparotomy. Timing risk factors considered were; age at presentation to the treating hospital with first symptoms of NEC, time from presentation with first symptoms to intervention, and transfer in to surgical centre*.* Operative findings considered were; sparing of at least one region of colon, and definitive diagnosis of SIP at time of operation. Markers of early morbidity considered were; need for second laparotomy and parenteral nutrition requirement at 28 days post-decision to intervene surgically.

All statistical analysis was performed using Stata version 13 (StataCorp. 2009. Stata: Release 13. Statistical Software. College Station, TX: StataCorp LP). LMSGrowth Excel add in version 2.77 (Pan H, Cole TJ. LMSGrowth, a Microsoft excel add-in to access growth references based on the LMS method. Version 2.77. <http://www.healthforallchildren.co.uk/;2012>) was used to calculate the birth weight z-scores.

**Supplementary table 1. Association of pre-operative characteristics, operative findings, choice of intervention, and early post-operative morbidities with mortality at one-year post-decision to intervene surgically.**

| **Characteristic** | | **Died**  n (%) | **Alive**  **n (%)^** | **OR (95% CI)** |
| --- | --- | --- | --- | --- |
| **Categorical antenatal/birth factors** | | | | |
| **White British**  (missing 20 infants) | Yes | 24 (45%) | 88 (65%) | 0.44 (0.2-0.9) p =0.01 |
|  | No | 29 (55%) | 47 (35%) |  |
| **Male** | Yes | 37 (63%) | 89 (62%) | 1.0 (0.5-2.1) p=0.90 |
|  | No | 22 (37%) | 55 (38%) |  |
| **Small for gestational age** | Yes | 14 (25%) | 16 (11%) | 2.6 (10.7-6.2) p=0.02 |
|  | No | 43 (75%) | 128 (89%) |  |
| **Multiple foetuses** | Yes | 15 (25%) | 34 (24%) | 1.1 (0.5-2.3) p=0.8 |
|  | No | 44 (75%) | 110 (76%) |  |
| **Mode of delivery** | Caesarean | 27 (46%) | 57 (40%) | 1.3 (0.7-2.5) p= 0.4 |
|  | Vaginal | 32 (54%) | 87 (60%) |  |
| **Antenatal corticosteroids** | Yes | 37 (76%) | 93 (73%) | 1.1 (0.5-2.7) p= 0.8 |
|  | No | 12 (24%) | 34 (27%) |  |
| **Maternal Indomethacin use** | Yes | 1 (2%) | 2 (2%) | 1.3 (0.02-25.0) p=0.8 |
|  | No | 44 (98%) | 114 (98%) |  |
| **Continuous antenatal/birth factors** | | | | |
| **Characteristic** | | **OR (95% CI)** | | |
| **Gestational age (per completed week increase)** | | 0.9 (0.88-1.02) p=0.15 | | |
| **Neonatal risk factors** | | | | |
| **APGAR<5 at 10 minutes** | Yes | 5 (11%) | 11 (9%) | 1.3 (0.3-4.3) p=0.7 |
|  | No | 41 (89%) | 115 (91%) |  |
| **Umbilical Catheter ever used** | Yes | 31 (74%) | 68 (65%) | 1.5 (0.7-3.8) p=0.3 |
|  | No | 11 (26%) | 37 (35%) |  |
| **Cardiac Anomaly** | Yes | 6 (10%) | 12 (8%) | 1.2 (0.4-3.8) p=0.7 |
|  | No | 53 (90%) | 132  (92%) |  |
| **Presence of a PDA** | Yes | 36 (62%) | 78 (54%) | 1.4 (0.7-2.7) p = 0.3 |
|  | No | 22 (38%) | 66 (46%) |  |
| **Indomethacin for PDA Closure** | Yes | 12 (22%) | 33 (24%) | 0.9 (0.4-2.0) p=0.8 |
|  | No | 42 (78%) | 104 (76%) |  |
| **PDA ligation performed** | Yes | 3 (5%) | 11 (8%) | 0.7 (0.1-2.6) p=0.5 |
|  | No | 55 (95%) | 133 (92%) |  |
| **Non-PDA Cardiac Surgery**  **(cornfield estimation)** | Yes | 0 | 5 (3%) | 0 (0-1.9) p = 0.15 |
|  | No | 59 (100%) | 139 (97%) |  |
| **Non-cardiac congenital anomaly** | Yes | 10 (17%) | 13 (9%) | 2.0 (0.8-5.6) p=0.1 |
|  | No | 48 (83%) | 131 (91%) |  |
| **Antacid use at presentation** | Yes | 7 (12%) | 9 (6%) | 2.0 (0.6-6.4) p=0.2 |
|  | No | 51 (88%) | 132 (94%) |  |
| **Ever enterally fed prior to diagnosis** | Yes | 51 (86%) | 121 (84%) | 1.2 (0.5-3.3) p=0.7 |
|  | No | 8 (14%) | 23 (16%) |  |
| **Formula milk at diagnosis** | Yes | 14 (24%) | 34 (24%) | 1.0 (0.5-2.1) p=1.0 |
|  | No | 45 (76%) | 110 (76%) |  |
| **Blood Transfusion <2 weeks prior to diagnosis** | Yes | 23 (41%) | 44 (32%) | 1.5 (0.7-2.9) p=0.25 |
|  | No | 33 (59%) | 92 (68%) |  |
| **Categorical presentation factors** | | | | |
| **Abdominal wall erythema or discolouration at presentation** | Yes | 25 (42%) | 40 (28%) | 1.9 (0.96-3.8) p=0.04 |
|  | No | 34 (58%) | 104 (72%) |  |
| **Inotropes at presentation** | Yes | 18 (31%) | 29 (20%) | 1.8 (0.8-3.7) p=0.1 |
|  | No | 40 (69%) | 114 (80%) |  |
| **Inotropes required at time of surgical intervention** | Yes | 32 (54%) | 43 (30%) | 2.8 (1.4-5.4) p =0.001 |
|  | No | 27 (46%) | 100 (70%) |  |
| **Ventilated at presentation** | Yes | 42 (71%) | 82 (57%) | 1.9 (0.9-3.8) p=0.06 |
|  | No | 17 (29%) | 62 (43%) |  |
| **Ventilated at time of surgical intervention** | Yes | 55 (93%) | 116 (81%) | 3.2 (1-13.1) p=0.03 |
|  | No | 4 (7%) | 27 (19%) |  |
| **Pre-operative radiological evidence of perforation** | Yes | 31 (53%) | 64 (44%) | 1.4 (0.7-2.7) p=0.3 |
|  | No | 28 (47%) | 80 (56%) |  |
| **Transferred in to surgical centre** | Yes | 40 (68%) | 112 (78%) | 0.6 (0.3-1.3) p=0.14 |
|  | No | 19 (32%) | 32 (22%) |  |
| **Continuous presentation factors** | | | | |
| **Characteristic** | | **OR (95% CI)** | | |
| **Age at presentation to the treating hospital with first symptoms of NEC (per day increase)** | | 0.98 (0.96-1.0) p =0.06 | | |
| **Time from presentation to decision to intervene (per day increase)** | | 0.96 (0.92-1.01) p = 0.14 | | |
| **Operative findings** | | | | |
| **At least one region of colon unaffected by NEC** | **Yes** | 43 (86%) | 137 (99%) | 0.04 (0.001-0.4) p= 0.0001 |
|  | **No** | 7 (14%) | 1 (1%) |  |
| **Definitive diagnosis of SIP at laparotomy** | **Yes** | 11 (21%) | 21 (15%) | 1.5 (0.6-3.6) p=0.3 |
|  | **No** | 41 (79%) | 118 (85%) |  |
| **Markers of early Morbidity** | | | | |
| **Need for Parenteral Nutrition at 28 days post-decision to intervene surgically** | **Yes** | 52 (88%) | 90 (62%) | 4.5 (1.8-12.4) p=0.0003 |
|  | **No** | 7 (12%) | 54 (38%) |  |
| **Need for second laparotomy** | **Yes** | 13 (22%) | 33 (23%) | 1.0 (0.4-2.1) p=0.9 |
|  | **No** | 46 (78%) | 111 (77%) |  |
